# Cervical Infection as a Novel Risk Factor for Perineal Obstetrical Trauma: A Cross-Sectional Study †

**DOI:** 10.3390/jcm14134477

**Published:** 2025-06-24

**Authors:** Natalia Katarzyna Mazur-Ejankowska, Maciej Ejankowski, Piotr Wąż, Anna Chyc-Myrmuła, Magdalena Emilia Grzybowska

**Affiliations:** 1 Clinic of Obstetrics and Gynecology, Gynecological Oncology and Endocrine Gynecology, University Clinical Centre, Debinki 7, 80-952 Gdansk, Poland; natalia.mazur@gumed.edu.pl (N.K.M.-E.); maciej.ejankowski@gumed.edu.pl (M.E.); 2 Department of Gynecology, Obstetrics and Neonatology, Medical University of Gdansk, Marii Sklodowskiej-Curie 3a, 80-210 Gdansk, Poland; 3 First Doctoral School, Medical University of Gdansk, Marii Sklodowskiej-Curie 3a, 80-210 Gdansk, Poland; 4 Department of Nuclear Medicine, Medical University of Gdansk, Marii Sklodowskiej-Curie 3a, 80-210 Gdansk, Poland; piotr.waz@gumed.edu.pl; 5 Student Scientific Circle of Gynecology and Obstetrics, Medical University of Gdansk, Marii Sklodowskiej-Curie 3a, 80-210 Gdansk, Poland; chyc.anna@gumed.edu.pl

**Keywords:** cervical infection, perineal trauma, vaginal childbirth, *group B Streptococcus* rectovaginal colonization, surgical site infection

## Abstract

**Background/Objectives:** Perineal obstetrical trauma sustained during vaginal delivery has a profound impact on female quality of life. The aim of the cross-sectional study was to analyze the association between active bacterial cervical infection and *group B Streptococcus (GBS)* rectovaginal colonization in the 35th–37th weeks of pregnancy with the degree of delivery perineal trauma. **Methods:** The study included 778 women after vaginal delivery. Maternal characteristics, including age, concomitant diseases, parity, obstetrical history, and cervical swab results conducted at admission and rectovaginal bacterial swabs at the 35th–37th weeks of pregnancy, were analyzed. The rates of perineal tears were compared between the physiological and pathological cervical swab groups and between the *GBS*-positive and *GBS*-negative colonization groups. **Results:** At admission to delivery, active cervical infection was diagnosed in 269 (35.9%) women. After vaginal delivery, 361 (49.3%) women had an intact perineum, and 288 (39.3%), 78 (10.7%), 4 (0.6%), and 1 (0.1%) had 1st–4th-degree perineal tears, respectively. Statistical analyses of the logistic regression model found that GBS colonization at the 35th–37th weeks of pregnancy (OR 1.56, *p* = 0.035) and pathological flora at admission (OR 1.54, *p* = 0.019) were associated with perineal tears. A higher vaginal parity was found to be a protective factor (OR 0.49, *p* < 0.000). **Conclusions:** High birthweight, longer second stage of labor duration, and primiparity were associated with increased rates of perineal trauma. Active cervical infection at admission and *GBS* colonization at the 35th–37th weeks of pregnancy were found to be risk factors for perineal tears. A protective factor for an intact perineum was a higher number of prior vaginal deliveries.

## 1. Introduction

Human microbiome studies continue to gather scientific interest, including the vaginal microbiome, which has been predominantly studied in correlation with preterm birth and premature rupture of the membranes (PROM).

No studies regarding the association between vaginal bacterial infection and rectovaginal GBS colonization and the risk of perineal tissue trauma sustained during labor have been conducted to date.

In surgery, for decades, wound infection and bacterial colonization were known to be risk factors for prolonged and complicated healing and wound dehiscence [1,2]. Numerous preventative measures are taken to eliminate wound infection and colonization, from adequate preparation of the operative field to antibiotics used empirically in high-risk infection sites [3,4].

In urology and urogynecology, the microbiome of the urinary bladder has been studied in detail to explore the consequences of chronic bacterial infection, colonization, and microbiota imbalance on the function of the organ. Long-term studies confirmed that urinary bladder bacterial chronic infection, colonization, and changes, such as a misbalance of the microbiome, may cause numerous conditions, including urinary incontinence [5,6], which has not been associated with microbiome changes until recent findings [7,8,9,10].

The vaginal microbiome has been extensively studied with regard to pregnancy complications, including preterm birth, PROM, intrauterine fetal growth restriction, and intrauterine infections [11,12,13]. An altered vaginal microbiome leads to the modulation of local immunity, resulting in abnormal production rates of pro-inflammatory factors such as cytokines, which are linked to the above-mentioned pregnancy complications.

Furthermore, numerous studies investigating the effects of the vaginal microbiome on the health of the neonate were conducted, proving the crucial role of vaginal delivery on the immunology of the newborn throughout their lifetime [14,15]. Recently, the placental microbiome has been investigated in correlation with possible adverse outcomes of pregnancy, although authors have published contrasting findings, and the subject remains controversial [16,17,18].

The studies of the vaginal microbiome during pregnancy have not taken into account comparing vaginal delivery outcomes of women with a normal vaginal microbiome to those with active vaginal infection or colonization. The authors hypothesized that bacterial infection and colonization of the cervix and vagina during delivery may be risk factors for perineal trauma, as the durability of tissues may be decreased, and hence, tissues may be more prone to mechanical damage.

## 2. Materials and Methods

A cross-sectional, retrospective study was designed, and approval was granted by the Bioethics Committee of the Medical University of Gdansk. The study was conducted in the tertiary obstetrics center. The average yearly rate of births conducted at the center is about 3500 deliveries. Pregnant women presenting to the emergency ward and labor ward of the Obstetrics Clinic of the tertiary center were consecutively enrolled in the study. At admission to the emergency department of the Obstetrics Clinic, a cervical and vaginal swab was conducted to establish the infection and colonization status of the enrolled group. The swabs were transported to the university laboratory, where aerobic and anaerobic bacterial and fungal cultures were cultivated, additionally screening for intracellular bacteria such as *Ureaplasma urealyticum* and *Mycoplasma hominis*. In the country where the study was performed, the national guidelines for gynecologists and obstetricians advise conducting a vaginal and rectal bacterial swab during the 35th–37th week of every pregnancy as a screening method for *group B Streptococcus* colonization. 

The study group consisted of women after vaginal delivery with 1st- to 4th-degree perineal tears, and the control group consisted of women after vaginal delivery with an intact perineum. The results of *GBS* swabs from the study and control groups were collected and noted at admission to the hospital. The data regarding the obstetrical history, the mode of delivery, including mechanical or pharmacological induction of labor, neonatal weight, perineal tear degree, and the patient’s concomitant diseases, such as pre-pregnancy diabetes mellitus, gestational diabetes mellitus, and chronic and gestational hypertension. Exclusion criteria included cesarean section or instrumental delivery, breech vaginal delivery, vaginal delivery with episiotomy, delivery of preterm infants under 37 weeks of gestation, and delivery of neonates weighing under 2000 g. Patients with incomplete medical records, such as those who were not screened for *GBS* colonization during pregnancy and those without a vaginal swab conducted at admission, were excluded from the study.

A statistical analysis of the gathered data was conducted after a cross-check for data correctness conducted by two authors separately, N. M-E. and M. E. The study included 778 women after vaginal delivery. Due to a lack of bacterial swab results, 46 women were excluded from further analyses, and 732 women were further analyzed. Participant age, height, weight, medical history, obstetrical data, and cervical swab results conducted at admission and at the 35th–37th weeks of pregnancy were analyzed. Perineal tear rates were compared between the groups with physiological and pathological cervical swabs and between the groups with *GBS*-positive and *GBS*-negative rectovaginal colonization. 

Continuous variables were expressed as the mean and standard deviation. Categorical variables were expressed as the percentages of the complete group; median, first quartile, and third quartile were reported for non-normally distributed populations. For quantitative variables analyses, the Shapiro–Wilk and Wilcoxon tests were used. The chi-squared test and Fisher’s exact test were used for categorical variables analysis. Multivariable stepwise logistic regression analysis with Akaike Information Criterion was performed to find predictors of perineal tear group membership to determine possible risk factors. The level of statistical significance was set at *p* < 0.05.

## 3. Results

The characteristics of the study group, including mean age and BMI, were 31.70 ± 4.72 years and 28.65 ± 4.63 kg/m^2^, respectively (Table 1). Out of 732 women, 361 women had an intact perineum, and 371 women had perineal tears in the 1st to 4th degrees.

The average fetal weight was significantly lower in the intact perineum group as compared to the group with perineal tears (median 3372.5 g versus mean 3481.3 g, *p* < 0.0001). The groups did not differ in terms of age and BMI. In the intact perineum group, 48 women (13.3%) were in their first pregnancy, compared to 103 (27.8%) in the perineal tear group. For 59 women (16.3%) in the intact perineum group, it was their first vaginal birth, compared to 130 women (27.8%) in the perineal tear group (*p*-value < 0.0001). PROM was confirmed in 45 (12.5%) and 42 (11.3%) of the females in the intact perineum group and perineal tears group, respectively (*p* > 0.05).

The groups did not differ in terms of the duration of the first stage of labor (*p* > 0.05). The second stage of labor was significantly longer in the perineal tears group (*p* < 0.02). The duration of the second stage of labor in the intact perineum group was <1 h in 322 women (89.2%), from 1 to 2 h in 34 women (9.4%), and over 2 h in 5 women (1.4%). In the perineal tears group, the duration of the second stage of labor was <1 h in 303 women (81.7%), from 1 to 2 h in 55 women (14.9%), and over 2 h in 13 women (3.5%) (Table 1).

Active cervical infection was diagnosed at hospital admission prior to delivery in 269 (35.9%) women, and physiological flora was diagnosed in 463 women (64.1%). After vaginal delivery, 361 (49.3%) women had an intact perineum, and 371 women (50.7%) had perineal tears, including 288 (39.3%), 78 (10.7%), 4 (0.6%), and 1 (0.1%) first- to fourth-degree perineal tears (Table 2).

In the physiological and pathological bacterial swab groups, 244 (52.7%) and 117 (43.5%) women had no perineal trauma, and 219 (47.3%) and 152 (56.5%) had perineal tears, respectively (*p* = 0.023) (Table 2). In the rectovaginal swab *GBS*-negative group, 235 (49.6%) had no tears and 239 (50.4%) had perineal tears, whereas in the rectovaginal *GBS*-positive group, 55 (38.5%) had no tears and 88 (61.5%) had perineal tears (*p* < 0.02) (Table 3).

Primiparity, longer second labor stage duration, and higher birthweight were significantly associated with perineal tears (*p* < 0.02). Maternal age, BMI, premature rupture of membranes, and labor preinduction did not differ between the intact perineum group and the perineal tears group. A higher number of prior vaginal deliveries was associated with no perineal trauma (*p* < 0.0001). 

When multivariable stepwise logistic regression was applied, *GBS* rectovaginal colonization at the 35th–37th weeks of pregnancy (OR 1.56, *p* = 0.035) and pathological cervical and vaginal flora at admission (OR 1.54, *p* = 0.019) were found to be risk factors for perineal tears. Vaginal parity in a model with one additional birth was a protective factor for intact perineum (OR 0.49, *p* < 0.000) (Table 4).

The chance of a nulliparous patient being in the perineal tear group is 2.04 times greater than for a patient with at least one previous vaginal delivery (Table 4). Another risk factor for perineal trauma was confirmed to be the birthweight of the neonate. When the birthweight was 1000 g higher than the initial 2000 g, the chance that the patient would belong to the perineal tears group was 2.36 times higher than for a patient with a neonatal birthweight of 2000 g.

The analyses of cervical and vaginal swabs conducted at admission to the hospital revealed that *Lactobacillus* spp. colonization was present in 463 patients, corresponding to 62.9% of the study population (Table 5). The most common cervical and vaginal pathogen in the study population was *Ureaplasma urealyticum*, present in 101 swabs, corresponding to 13.7% of the study population. *Streptococcus agalactiae* (*GBS*) colonization was found in 74 swabs, corresponding to 10.1% of the study group. A detailed analysis of cervical and vaginal swabs of the study population is presented in Table 5.

## 4. Discussion

The study confirms that primiparity, high birthweight, and a longer second stage of labor duration are associated with increased rates of perineal trauma, as has been frequently reported in previous international studies on the population of pregnant patients undergoing vaginal delivery. The aim of the study was to investigate the association between *GBS* rectovaginal colonization at the 35th–37th weeks of pregnancy and ongoing cervical/vaginal infection during admission to the delivery ward and perineal tears sustained during vaginal childbirth. 

The motivation and inspiration to conduct the study arose through clinical practice, as the authors and our team of medical practitioners observed a higher level of perineal tissue trauma sustained during vaginal labor in patients with symptoms of vaginal infection. Infected tissues, due to changes in the pH, edema, and, on a microscopical level, through the weakening of the cell wall, are more prone to mechanical damage [19]. The toxins that are produced by bacteria and fungi have a cytotoxic effect and damage the normal structure and durability of the cells and tissues [20,21]. The above hypothesis was not found to have been investigated to date in relation to vaginal birth perineal trauma.

Maternal rectovaginal *GBS* colonization was proven to cause numerous diseases, including maternal sepsis, prenatal complications of stillbirth, and neonatal complications, including preterm delivery, hypoxic encephalopathy, and early, late, and very late onset neonatal *GBS* disease, which may lead to sepsis of the neonate [22].

Maternal *GBS* colonization has been internationally investigated, and the worldwide estimate for maternal *GBS* colonization is at 18% with regional variation [23]. The lowest prevalence of *GBS* colonization is noted in Southern and Eastern Asia regions, and it corresponds directly with the lower *GBS* neonatal disease incidence in Asia [23]. The prevalence of maternal *GBS* was highest in the Caribbean (34%) and lowest in Melanesia (2%) [23]. Europe, North America, and Australia have a similar prevalence of maternal rectovaginal *GBS* colonization, which is estimated at 15–21% of the pregnant population [23].

In Poland, *GBS* rectovaginal maternal colonization is estimated at 19%, ranging between 8% and 27% [24], which corresponds with data from our single-center study that confirmed *GBS* colonization in 23% of women at the 35th–37th weeks of gestation. The incidence of *GBS* colonization at admission was at 10.1% (Table 5). The difference between *GBS* colonization rates is probably due to both vaginal and rectal swabs, which are taken at the 35th–37th weeks of gestation, as it is a national standard in Poland, as opposed to only taking a cervical/vaginal swab at admission to the hospital in the study group.

Interestingly, no data regarding bacterial colonization as a risk for perineal trauma sustained during childbirth was found upon extensive research of the literature. However, *GBS* colonization has, to date, been widely researched with regard to maternal infections, including sepsis, meningitis, pneumonia, and urinary tract infections in pregnant and postpartum women [25]. No studies regarding rectovaginal *GBS* colonization and the risk of perineal lacerations and infection rates were found by the authors.

In surgical fields, more in-depth investigations of the microbial spectrum causing wound dehiscence were conducted [26,27,28]. The detection of Gram-positive pathogens, especially *Enterococcus* spp., was found to be an independent risk factor for reduced wound healing, and empirical antibiotic coverage was found to be advantageous for high-risk patients [27].

Turtiainen et al. conducted a study to uncover the relationship between surgical wound bacterial colonization and the development of surgical site infection (SSI) after lower limb vascular surgery [28]. Most SSIs in vascular surgery are caused by *Staphylococcus* spp., which are part of the normal skin flora. Swabs for microbiological analyses were taken from surgical wounds at four different time intervals: before surgery, just before the surgical area had been scrubbed, at the end of surgery, and on the first and second postoperative days. The most common bacteria isolated were *coagulase-negative Staphylococcus* spp. (80%), *Corynebacterium* spp. (25%), and *Propionibacterium* spp. (15%). In 13 (62%) cases, the same bacterial isolates were found in the perioperative study samples as in the infected wounds. The incidence of SSI was 21%. Multivariate analysis revealed that high bacterial load on the second postoperative day and diabetes independently increased the risk of SSI. Elective redo surgery was protective against the development of SSI. The authors concluded that a high bacterial load in the postoperative surgical wound independently increases the risk of the development of SSI.

In line with the study, several studies were conducted confirming that the increasingly obese obstetrical population had an increased frequency of CS and increased vulnerability to SSI [29,30]. The microbiome at the site of skin incision before and after CS was established through bacterial swabs. It was found that in obese women, the incision site harbors a significantly higher bacterial biomass with a lower diversity. The findings suggest the microbiota at the incision differs between obese and non-obese pregnant women and changes throughout CS. An interaction between vaginal and cutaneous dysbiosis at the incision site may explain the initially increased risk for SSI among obese pregnant women. Unfortunately, no similar studies were conducted on the population of pregnant women during and after vaginal childbirth.

### 4.1. Future Directions

Investigating bacterial infection as a risk factor for perineal trauma sustained during vaginal delivery encourages the consideration of any action that could be taken prior to vaginal delivery to decrease the risk of infection.

Historically, the female reproductive tract was believed to be a sterile environment, yet upon investigation, it was proven that *Lactobacillus* spp. vaginal colonization is present and helps to maintain an acidic pH. Furthermore, colonization helps with fertilization by promoting epithelial barrier function [31].

Altering the vaginal microbiome could hold the answer to lowering the rate of vaginal infections and decreasing the rate of preterm births. The vaginal microbiome has been associated with triggering spontaneous preterm birth through modulation of local immunity [32]. Immunophenotyping of the vaginal microbiome in pregnant populations led to categorizing patients into immune subtypes based on local immune status and naming certain subtypes as more susceptible to preterm birth [32]. Amabebe et al. investigated the antimicrobial properties of vaginal *Lactobacillus crispatus* against preterm birth-associated bacteria. An in vitro study was conducted in which *Lactobacillus crispatus* inhibited the growth of *Gardnerella vaginalis*, one of the most common pathogens associated with preterm delivery induced by an inflammatory reaction [33]. Perhaps by introducing *Lactobacillus crispatus* to the vaginal microbiome early during the pregnancy or prior to conception, a reduction in pathogenic bacterial growth would be observed, leading to a decrease in vaginal infection rate during labor. Further in vitro and in vivo studies of the vaginal microbiome are needed to evaluate the impact of altering the microbiome through oral and topical supplementation.

To translate our findings into clinical practice, validation studies in more diverse populations are necessary. Establishing categories of the vaginal microbiome associated with a greater risk of intrapartum complications could lead to introducing timely interventions such as immunomodulatory therapies.

### 4.2. Study Limitations and Strengths

The main limitation of the study is conducting only one per-patient cervical/vaginal bacterial swab prior to delivery. It is possible that by relying upon a single bacterial swab, especially performed under time pressure due to ongoing labor, the results may be inaccurate. Additionally, the rectovaginal swabs taken during the 35th–37th weeks of gestation were taken in outpatient clinics and sent to numerous laboratories in the district, which could have led to inaccurate results due to different laboratory standards. Finally, the cervical/vaginal swab did not include viral pathogens such as human papillomavirus and some bacterial species, for example, *Chlamydia trachomatis* and *Neisseria gonorrhoeae*. Finally, a lack of previous similar studies to compare our study to could be viewed as a limitation. 

The strengths of the study are the large study and control groups and multivariate statistical analysis to eliminate bias. The high standard of the reference university laboratory conducting the vaginal swabs at admission, with attention to *Ureaplasma* spp. and *Mycoplasma* spp., should be underlined.

## 5. Conclusions

High birthweight, longer second stage of labor duration, and primiparity were associated with increased rates of perineal obstetrical trauma, as has been frequently proved in previous studies on the population of pregnant patients undergoing vaginal childbirth. For the first time to date, it was investigated and concluded in the study that *GBS* rectovaginal colonization at the 35th–37th weeks of pregnancy and active vaginal bacterial infection at admission to the delivery ward were found to be associated with perineal tears sustained during childbirth. In line with previous studies conducted on the pregnant population, a higher number of prior vaginal deliveries was found to be a protective factor for an intact perineum.

The authors are in the process of designing a clinical trial to further investigate the influence of cervical and rectovaginal microbiomes on the results of vaginal childbirth with regard to perineal trauma. More studies are urgently needed to further investigate this subject with different populations and in larger and more diverse patient groups.

## Figures and Tables

**Table 1:** Characteristics of the study groups.

Variables	Intact Perineum (n = 361)	Perineal Tear 1st–4th Degrees (n = 371)	*p*-Value
**Age (years)**			0.48 ^f^
<19	0	1 (0.3%)	
19–29	110 (30.5%)	119 (32.1%)	
30–39	234 (64.8%)	227 (61.2%)	
≥40	17 (4.7%)	24 (6.5%)	
**BMI (kg/m^2^)**			0.14 ^f^
<18.5	0	1 (0.3%)	
18.5–24.99	49 (13.6%)	66 (1.8%)	
25–29.99	187 (51.8%)	172 (92.0%)	
30–34.99	89 (24.7%)	107 (28.8%)	
35–39.99	25 (6.9%)	19 (5.1%)	
≥40	11 (3.1%)	6 (0.2%)	
**BMI median (kg/m^2^)**	28 (26; 31)	28 (25; 31)	0.23 ^w^
**Fetal weight (grams)**	3372.5 (3000; 3637.5)	3481.3 ± 446.8	**<0.0001 ^w^**
**Pregnancy number**			**<0.0001 ^f^**
1st	48 (13.3%)	103 (27.8%)	
2nd	149 (41.3%)	153 (41.2%)	
3rd	81 (22.4%)	69 (18.6%)	
4th	33 (9.1%)	35 (9.4%)	
5th	30 (8.3%)	7 (1.9%)	
≥6th	20 (5.5%)	4 (1.1%)	
**Vaginal birth number**			**<0.0001 ^f^**
1st	59 (16.3%)	130 (35.0%)	
2nd	182 (50.4%)	183 (49.3%)	
3rd	78 (21.6%)	40 (10.8%)	
4th	24 (6.6%)	14 (3.8%)	
5th	10 (2.8%)	2 (0.5%)	
≥6th	8 (2.2%)	2 (0.5%)	
**Premature rupture of membranes (PROM)**			0.25 ^c^
No n = 645	316 (87.5%)	329 (88.7%)	
Yes n = 87	45 (12.5%)	42 (11.3%)	
**First stage of labor**			0.14 ^c^
<3 h	118 (32.7%)	104 (28.0%)	
3–6 h	171 (47.4%)	177 (47.7%)	
6–9 h	57 (15.8%)	61 (16.4%)	
≥9 h	15 (4.2%)	29 (7.8%)	
**Preinduction of labor**			0.96 ^c^
**Foley catheter**	63 (17.5%)	63 (17.0%)	
**Induction of labor**			0.42 ^c^
**Oxytocin**	97 (26.9%)	89 (24.0%)	
**Concomitant diseases**			0.09 ^f^
No hypertension	332 (92.0%)	346 (93.3%)	
Gestational hypertension (GH)	20 (5.5%)	23 (6.2%)	
Chronic hypertension	9 (2.5%)	2 (0.5%)	
**Concomitant diseases**			0.91 ^f^
No diabetes	314 (87.0%)	320 (86.3%)	
Gestational diabetes (GDMG1)	16 (4.4%)	16 (4.3%)	
Gestational diabetes (GDMG2)	27 (7.5%)	32 (8.6%)	
Diabetes mellitus (DM)	4 (1.1%)	3 (0.8%)	
**Previous cesarian section**			0.25 ^c^
No	337 (93.4%)	357 (96.%)	
Yes	24 (6.7%)	14 (3.8%)	
**Second stage of labor**			**0.02 ^f^**
<1 h	322 (89.2%)	303 (81.7%)	
1–1.5 h	25 (6.9%)	37 (10.0%)	
1.5–2 h	9 (2.5%)	18 (4.9%)	
≥2 h	5 (1.4%)	13 (3.5%)	
**GBS colonization 35th** *–* 37th weeks: rectovaginal *			0.025 ^a^
Negative n = 474 (76.82%)	235 (81.0%)	239 (73.1%)	
Positive n = 143 (23.18%)	55 (19.0%)	88 (26.9%)	
**At admission:**			0.023 ^a^
Physiological swab	244 (67.6%)	219 (59.0%)	
Pathological swab	117 (32.4%)	152 (41.0%)	

^c^—Pearson’s Chi^2^ test with Yates’ continuity correction, ^f^—Fisher’s exact test for count data, ^w^—Wilcoxon test, ^a^—χ^2^ test. * Patients with incomplete data or without swab results were excluded from the group.

**Table 2:** Distribution of the perineal tears in the physiological and pathological swab groups.

Variables	Physiological Swab (n = 463)	Pathological Swab (n = 269)	*p*-Value
**Perineal tear degree**			**0.012 ^f^**
No tear (n = 361)	244 (52.7%)	117 (43.5%)	
1st (n = 288)	163 (35.2%)	125 (46.5%)	
2nd (n = 78)	54 (11.7%)	24 (8.9%)	
3rd (n = 4)	2 (0.4%)	2 (0.7%)	
4th (n = 1)	0	1 (0.4%)	
**Perineal tear**			**0.023 ^a^**
No tear	244 (52.7%)	117 (43.5%)	
Tears: 1st, 2nd, 3rd, 4th	219 (47.3%)	152 (56.5%)	

^f^—Fisher’s exact test for count data, ^a^—χ^2^ test.

**Table 3:** Comparison between perineal tears and *GBS* colonization groups.

Variables	No *GBS* Colonization at the 35th–37th Weeks n = 474 *	*GBS* Colonization at the 35th–37th Weeks n = 143 *	*p*-Value
**Perineal tear degree**			**0.017 ^f^**
No tear	235 (49.6%)	55 (38.5%)	
1st	180 (38.0%)	73 (51.1%)	
2nd	56 (11.8%)	13 (9.1%)	
3rd	3 (0.6%)	1 (0.7%)	
4th	0	1 (0.7%)	
**Perineal tear**			**0.025 ^a^**
No tear	235 (49.6%)	55 (38.5%)	
Tears: 1st, 2nd, 3rd, 4th	239 (50.4%)	88 (61.5%)	

^f^—Fisher’s exact test for count data, ^a^—χ^2^ test. * Patients with incomplete data or without swab results were excluded from the group.

**Table 4:** Logistic regression model with a stepwise algorithm comparing patients with intact perineum (reference group) to patients with perineal tears (variable).

Variable	Estimate	Standard Error	Z Value	*p*-Value	Odds Ratio
(Intercept)	−1.6201	0.6722569	−2.410	0.016	
Z1—*GBS* colonization in the 35th–37th weeks of pregnancy	0.4450	0.2113007	2.106	0.035	1.5604
Z2—Cervical swab at admission	0.4345	0.1846144	2.354	0.019	1.5442
Z3—Vaginal birth	−0.7197	0.1060768	−6.785	<0.00001	0.4869
Z4—Birthweight	0.0009	0.0001945	4.425	0.00001	1.0009, 2.3641

Z1—*GBS* colonization in the 35th–37th weeks of pregnancy, a positive swab compared with a negative swab. Z2—Cervical swab at admission, the pathological swab group compared with the physiological swab group. Z3—Vaginal birth compared with one additional birth. Z4—Birthweight compared with a weight increase of 1 g or a weight increase of 1000 g.

**Table 5:** Cervical and vaginal swab results taken at admission to the tertiary center.

Bacterial Species Cultured from Cervical/Vaginal Swab at Admission	Number of Patients (n = 732)	Percentage of the Study Population	Gram Positive (+), Gram Negative (−), or Fungi (F)
*Lactobacillus* spp.	463	62.9%	+
*Ureaplasma urealyticum*	101	13.7%	+
*Streptococcus agalactiae (GBS)*	74	10.1%	+
*Candida* spp.	32	4.3%	F
*Enterococcus faecalis*	22	3.0%	+
*Escherichia coli*	18	2.4%	−
*Staphylococcus haemolyticus*	9	1.2%	+
*Klebsiella* spp.	6	0.8%	−
*Mycoplasma* spp.	5	0.7%	+
*Proteus mirabilis*	1	0.2%	−
*Staphylococcus epidermidis*	1	0.2%	+

## Data Availability

The original contributions presented in this study are included in the article. Further inquiries can be directed to the corresponding author(s).

## Abbreviations

GBS	*Group B Streptococcus*
OR	odds ratio
BMI	body mass index
PROM	premature rupture of the membranes
SSI	surgical site infection
CS	cesarean section
OB/GYN	obstetrics and gynecology
